# Maternal methyl supplemented diets and effects on offspring health

**DOI:** 10.3389/fgene.2014.00289

**Published:** 2014-08-26

**Authors:** Rachel J. O'Neill, Paul B. Vrana, Cheryl S. Rosenfeld

**Affiliations:** ^1^Department of Molecular and Cell Biology, University of ConnecticutStorrs, CT, USA; ^2^Institute for Systems Genomics, University of ConnecticutStorrs, CT, USA; ^3^Peromyscus Genetic Stock Center, University of South CarolinaColumbia, SC, USA; ^4^Department of Biological Sciences, University of South CarolinaColumbia, SC, USA; ^5^Department of Biomedical Sciences, Bond Life Sciences Center, University of MissouriColumbia, MO, USA; ^6^Bond Life Sciences Center, University of MissouriColumbia, MO, USA; ^7^Genetics Area Program Faculty Member, Bond Life Sciences Center, University of MissouriColumbia, MO, USA

**Keywords:** DNA methylation, diet, DOHaD, maternal, *in utero*, nutrition, nutriceutical

## Abstract

Women seeking to become pregnant and pregnant women are currently advised to consume high amounts of folic acid and other methyl donors to prevent neural tube defects in their offspring. These diets can alter methylation patterns of several biomolecules, including nucleic acids, and histone proteins. Limited animal model data suggests that developmental exposure to these maternal methyl supplemented (MS) diets leads to beneficial epimutations. However, other rodent and humans studies have yielded opposing findings with such diets leading to promiscuous epimutations that are likely associated with negative health outcomes. Conflict exists to whether these maternal diets are preventative or exacerbate the risk for Autism Spectrum Disorders (ASD) in children. This review will discuss the findings to date on the potential beneficial and aversive effects of maternal MS diets. We will also consider how other factors might influence the effects of MS diets. Current data suggest that there is cause for concern as maternal MS diets may lead to epimutations that underpin various diseases, including neurobehavioral disorders. Further studies are needed to explore the comprehensive effects maternal MS diets have on the offspring epigenome and subsequent overall health.

## Introduction

Mounting evidence suggests that developmental exposure to extrinsic factors (e.g., diet, chemical exposure, and stress) leads to pronounced epigenetic and phenotypic effects, commonly referred to as the “developmental origin of health and disease (DOHaD)” (Barker, 1997; Hanson and Gluckman, 2011; Barouki et al., 2012; Rosenfeld, 2012). Epigenetic changes are meiotically and mitotically heritable and include DNA methylation, histone protein modifications, chromatin re-arrangement, and non-coding RNAs (Rosenfeld, 2010).

**Figure 1:**
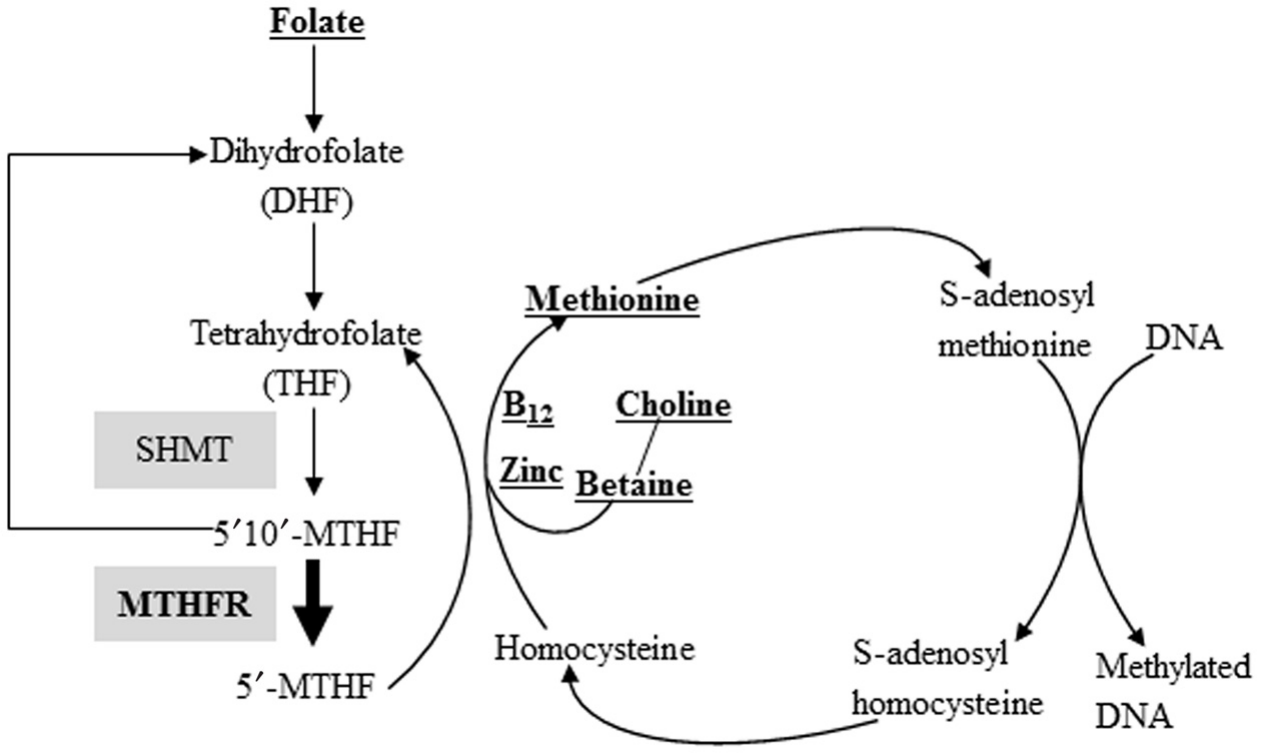
**Nutritional methyl-supplements and the DNA methylation reaction**. Methyl groups, either synthesized via the folate cycle or donated directly from betaine, are activated by the methyl activation cycle. Activated methyl groups are used to methylate cytosine residues of DNA. Both vitamin B_12_ and zinc are cofactors used in the transfer of methyl groups. Compounds listed in bold and underlined are methyl donors or cofactors included in various methyl supplemented diets. Select enzymes involved in this pathway, including MTHFR, where single nucleotide polymorphisms (SNP) variant genotypes have been documented (as detailed in the manuscript) are shaded.

Methylation of CpG sites is a potent regulator of gene expression, and this process is affected by various external, including dietary, factors. There has been growing interest in the potential protective effects that dietary supplements might exert on these epigenetic marks. Such nutraceuticals include molecules that directly lend methyl donors in the methyl activation cycle or serve as cofactors in these pathways, such as methionine, pyridoxine (vitamin B6), folate (vitamin B9), betaine (trimethylglycine), choline, Vitamin B_12_, and zinc (Figure 1). Many of these compounds are included in periconceptional/ prenatal vitamins, due to their ability to prevent neural-tube and other CNS defects in developing fetuses and other health benefits. While all of these factors exert key roles, emphasis has been placed on folic acid, wherein women are recommended to consume a multivitamin containing 0.8 mg of folic acid 1 month prior to conception and throughout the first few months of pregnancy (http://www.uspreventiveservicestaskforce.org/uspstf/uspsnrfol.htm; Wolff et al., 2009a,b; Steenblik et al., 2011).

Animal model studies have examined how maternal methyl supplemented (MS) diets impact the epigenome and/or phenotypic outcomes in developing offspring (Table 1A). Some of these studies have reported favorable effects of these diets on overall health. Other studies, though, have reported detrimental maternal MS alterations. Limited studies have examined linkages between maternal MS diets and epimutations/disease in her children (Table 1B).

**Table 1A:** **Animal model studies linking maternal MS diets and effects on offspring health and disease**.

**Publication**	**Animal model**	**Dosing regimen**	**Major findings**
Dolinoy et al., 2007	*a/a* and *A^vy^/a* mice	Two weeks prior to mating and throughout gestation and lactation, *a/a* female mice carrying *A^vy^/a* mice offspring were fed an AIN93G, AIN93G + bisphenol A (50 mg BPA/kg feed weight) or AIN 93G diet supplemented with BPA (50 mg /kg feed weight) and methyl supplemented (MS, including 4.3 mg of folic acid/kg feed weight, 0.53 mg of vitamin B12/kg feed weight, 5 g of betaine/kg feed weight, and 7.97 g of choline chloride/kg feed weight) diet.	Those females exposed to the BPA diet alone were reported to give birth to greater percentage of yellow coat color *A^vy^/a* offspring, who are prone to developing metabolic disorders. In contrast, females exposed to the combined diet gave birth to equivalent percentage of brown to yellow coat color *A^vy^/a* offspring, suggesting that the maternal MS diet abolished the harmful effects of BPA in this epigenetic animal model.
Wolff et al., 1998; Cooney et al., 2002	*a/a* and *A^vy^/a* mice	The a/a dams were placed on a control (NIH-31 diet) 3SZM diet 2 weeks prior to mating to *A^vy^/a* males and through parturition. Those receiving the methyl supplemented diet were then divided into those that received the MS diet for 1 week after birth followed by a lower dose of methyl supplements (HS diet) for the second week after birth and the NIH-31 thereafter. Other dams were placed on the MS diet 2 weeks prior to mating, HS diet for 1 week after birth followed by the NIH-31 thereafter. Methyl components in the 3SZM diet included: 15 g choline, 15 g betaine, 15 mg folic acid, 1.5 mg vitamin B12, 7.5 mg L-methionine, and 150 mg zinc. MS diet included 5 g choline, 5 g betaine, 5 mg folic acid, and 0.5 mg vitamin B_12_. Those in the HS diet included: 2.5 g choline, 2.5 g betaine, 2.5 mg folic acid, and 0.25 mg vitamin B_12_.	The combined studies indicated that maternal MS of a/a mice carrying *A^vy^/a* offspring increases the DNA methylation status of long terminal repeat (LTR) controlling *agouti* (*A*) expression and thereby, increasing the percentage of pseudoagouti coat color relative to yellow coat color *A^vy^/a* offspring with the former generally remaining healthy throughout their lifespan.
Waterland and Jirtle, 2003	*a/a* and *A^vy^/a* mice	The a/a females were placed on a control (NIH-31) or NIH-31 diet supplemented with methyl donors/cofactors (same as in Wolff et al., 1998) 2 weeks prior to mating to *A^vy^/a* males and throughout gestation and lactation.	These finding supported the earlier work of Wolff et al., 1998; Cooney et al., 2002 in that that it showed that MS to *a/a* carrying *A^vy^/a* offspring increased the percentage of pseudoagouti offspring by increasing the CpG methylation of the *A^vy^* locus.
Cropley et al., 2012	*a/a* and *A^vy^/a* mice	Two weeks prior to breeding and through weaning of the F_5_ generation, *A^vy^/a* males and *a/a* females were placed on a control (NIH-31) or this diet supplemented with methyl donors/cofactors: 15 g choline, 15 g betaine, 7.5 g L-methionine, 150 mg ZnSO_4_, 15 mg folic acid, and 1.5 mg vitamin B_12_. From weaning of F_5_ generation through F_8_ generation, all animals were placed on a control (NIH-31) diet.	MS increased proportion of pseudoagouti offspring through the F_5_ generation. However, removal of the MS diet in the F_5_ generation resulted in a return to the distribution of pseudoagouti to brown coat color *A^vy^/a* mice, suggesting that the epigenetic status of the germline of *A^vy^* are reversible after MS removal.
Li et al., 2011	C57Bl6J	Two weeks prior to mating initial pair through six generations, C57Bl6J males and females were placed either on a NIH-31 control diet or this diet supplemented with methyl donors/cofactors: 15 g choline, 15 g betaine, 7.5 g L-methionine, 150 mg zinc, 15 mg folic acid, and 1.5 mg vitamin B_12_.	MS increased the stochastic epigenetic variation in several loci with the effects becoming more pronounced in later generations.
Billington et al., 2013	Mice deficient for twisted gastrulation *(Twsg1)*	Females carrying mice deficient in this gene were fed a control (NIH-31) or MS diet that included folate, vitamin B_12_, choline, and betaine (Harlan Teklad-TD.01308) 2 weeks prior to mating and throughout gestation and lactation.	These transgenic deficient mice generally demonstrate midline facial and jaw defects. However, maternal MS exposure prevented the jaw but not midline defects in these mice.
Delaney et al., 2013	ApoE homozygous mutant mice	Females carrying these transgenic mutant mice were fed a control or MS (11.8 g/kg L-methionine, 13.5 mg/kg folic acid, 1560 mg/kg B_12_, 0.72 g/kg ZnS0_4_.7H_2_0, 0.015 g/kg betaine, and 19.4 g/kg choline chloride) diet 3 weeks prior to mating and throughout gestation and lactation. At weaning, all males were placed on a 42% high fat diet (HFD) until they were euthanized.	The maternal MS exposure alleviated the atherosclerosis that would otherwise occur in these mice. Correspondingly, those exposed to the diet regimen had inhibition of T lymphocyte expression of *Ccr2*, reduction in other inflammatory cytokines, and increased circulating HDL to LDL ratio.
Ash et al., 2014	Ts65Dn female mice (a mouse model for down syndrome and Alzheimer's disease) and C57/Bl6J Eicher X C3H/HeSnJ F1 male mice.	Breeder pairs were randomly assigned to a control chow-based diet (AIN-76A containing 1.1 g/kg choline chloride or a choline-supplemented diet (AIN-76A with 5.0 g/kg choline chloride). Weaned pups were placed on the control diet.	• Spatial mapping was compromised in control Ts65Dn mice relative normal disomic (2N) littermates. However, maternal choline supplementation improved spatial mapping of Ts65Dn mice.
			• Ts65Dn mice possessed decreased number and density of medial septal hippocampal basal forebrain cholinergic neurons (BFCNs) in control Ts65Dn mice relative disomic littermates. Maternal chonine supplementation increased the numbers of these neurons.
Medici et al., 2014	C3H and tx-j mice	C3H control females carrying tx-j conceptuses were fed 2 weeks prior to mating and throughout gestation (GD17) a control (8 mmol/kg choline of diet) or choline supplemented (36 mmol/kg choline of diet). Other foster C3H females received these same diets that extended through lactation (21 days postpartum).	Maternal choline supplementation of C3H dams prevented transcriptional deficits and reduced body weight at weaning observed in their tx-j offspring.
Carlin et al., 2013	C57BL/6J mice	Bred female mice were exposed to a control (18.5% protein, 12% fat and 69.5% carbohydrate), methyl supplemented (15 g choline chloride,15 g betaine [anhydrous], 15 mg folic acid, 1.5 mg vitamin B_12_, 7.5 g L-methionine, and 150 mg zinc from ZnSO_4_/7H_2_O), high fat (HF, 18.5% protein, 60% fat, and 20.5% carbohydrate) diet alone, or a combination of a HF and MS diet (maternal obesogenic methyl-supplemented, MOM diet) throughout gestation and lactation.	The methyl supplements in this diet regimen mitigated the harmful metabolic, behavioral, DNA methylation, and gene expression effects of the high fat component.
Downing et al., 2011	C57BL/6J mice	Two weeks before mating, dams were placed on an NIH-31 control diet or the 3SZM diet (Wolff et al., 1998) containing 15 g choline, 15 g betaine, 15 mg folic acid, 1.5 mg Vitamin B_12_, 7.5 g L-methionine, and 150 mg zinc. Control and MS dams were treated with 5.8 g/kg ethanol on gestational day 9.	• Gestational exposure to ethanol reduced offspring expression of *Igf2*.
			• Prior and concurrent exposure to a maternal MS diet abolished these ethanol-induced alterations in Igf2 expression.
			• Maternal MS decreased prenatal mortality, enhanced prenatal growth, and reduced the incidence of digit malformations.
Giudicelli et al., 2013	Sprague Dawley Rats	Female rats were fed a control or MS (12 g/kg L-methionine, 15 g/kg choline, 15 g/kg betaine, 1000 mg/kg vitamin B_12_, 15 mg/kg folic acid, and 180 mg/kg zinc) diet 21 to 28 prior to mating and throughout gestation and lactation.	• A maternal MS diet decreased circulating concentrations of leptin and led to hypermethylation of the *Ob* promoter in exposed offspring.
			• Exposed male offspring showed increased long-term body weight gain.
			• Exposed male and female offspring had suppressed post-natal growth.
Tsang et al., 2012	CD1 Mice	Pregnant mice were fed a control or 11mg/kg folate supplemented diet. Some members of both groups were then subjected from gestational day (GD)1 to GD18 to water containing Arsenic (As, 85 ppm).	Mouse fetuses at GD18 exposed to a maternal diet containing arsenic and MS demonstrated more pronounced hepatic DNA methylation changes and impaired fetal development and body weight gain compared to those exposed to the maternal arsenic-supplemented diet alone.
Huang et al., 2014	C57BL/6J Mice	Female mice were placed on a control folic acid 2 mg folic acid/ kg diet, AIN93G) diet, or this same diet with the recommended folic acid supplement (5 mg folic acid/kg diet, RF0IS) or high folic acid (40 mg folic acid/kg diet, HFoIS) before and after pregnancy. At birth, all dams were switched to the AIN93G control diet, and male weanling pups were placed on this same diet. Some male offspring were euthanized at 7 weeks of age; whereas, the remaining males per group were switched to a high fat diet until they were euthanized at 15 weeks of age.	• No differences were detected in males from the various groups euthanized prior to being exposed a high fat diet.
			• After 8 weeks of being on the high fat diet, males derived from dams on the HfoIS diet had increased incidence of obesity, glucose intolerance, and insulin resistance relative to controls.
			• HfoIS sons exhibited reduced serum adiponectin concentrations and corresponding decrease in adiponectin (*AdipoQ*) mRNA expression but increased global DNA hypermethylation in white adipose tissue compared to control males.
Schaible et al., 2011	C57BL/6J Mice	Females were exposed to a control (NIH-31) or this diet supplemented with methyl components (5 mg/kg folic acid; 0.5 g/kg vitamin B12; 5 g/kg betaine; and 5.76 g/kg choline [TD#01308, Harlan-Teklad]) 2 weeks prior to mating and throughout gestation and lactation.	A maternal MS diet led to:
			• Harmful and persistent changes in the offspring gut microbiota.
			• Offspring colonic mucosal DnA methylation and gene expression changes.
			• Increased incidence of colitis in exposed compared to control offspring.
Hollingsworth et al., 2008	C57BL/6J mice	Two weeks prior to mating and throughout pregnancy, females were placed on an NIH-31 control diet or AIN93G diet supplemented with methyl donors and cofactors (folic acid, Vitamin B_12_, choline chloride, betaine, and ZnSO_4_ X 7H_2_0 for a low methyl diet and a high methyl diet concentrations not specified in paper and reference Supplemental Table 1 is not included on the journal's website). F1 progeny were placed on the NIH-31 control diet	Maternal methyl supplementation increased the incidence of allergic airway disease in her offspring with corresponding hypermethylation of *Runx3*, a gene that has previously been shown to suppresses allergic airway disease.
Delaney et al., 2013	C57BL/6J mice	Three weeks prior to mating, females were placed on a NIH-31 control or a maternal methyl-supplemented diet (13.5 mg/kg folic acid, Vitamin B_12_ 1.56 g/kg, 15 mg/kg betaine, and 19.4 g/kg choline chloride). Weaned pups were either euthanized at 28 days of age or placed on the control diet	• F1 offspring exposed to the maternal MS were smaller in size at 28 days of age compared to controls.
			• T-cells from the maternal MS offspring possessed increased DNA methylation relative to those from control mice.
			• T cells from maternal MS mice exhibited reduced T cell chemokine receptor (*Ccr2* and *Ccr5*), C-X-C- chemokine receptor 3, *Tnfa*, and *Il4* gene expression and associated decreased chemotaxis.
Davison et al., 2009	Sprague Dawley Rats	Female rats were fed a control (8 mmol/kg choline chloride), choline-supplemented (36 mmol/kg choline chloride), or choline-deficient (0 mmol/kg choline chloride) diet from GD11 to GD17	A maternal MS (choline) or choline deficient diet resulted in selective epimutations in DNA methylation and histone protein marks.
Barua et al., 2014	C57Bl6J	Gestational and post-natal exposure (in case of pup assessments) to control diet, 0.4 mg folic acid-supplemented diet (lower dose), or 4 mg/kg folic acid-supplemented diet (higher dose)	Higher folic acid-supplemented diet led to:
			• Disruptive gene and protein expression changes in the cerebral hemispheres of newborn pups
			• Behavioral abnormalities in neonatal pups, including increased ultrasonic vocalizations, anxiety-like, and hyperactive behaviors.

**Table 1B:** **Human epidemiological studies linking maternal MS diets and effects on offspring health and disease**.

**Publication**	**Study population/design**	**Major findings**
Boeke et al., 2012	Determination of various maternal MS, including choline, and offspring umbilical cord methylation.	Increased maternal intake during the periconceptional period of choline was inversely correlated with umbilical cord methylation in sons but not daughters.
Haggarty et al., 2013	Measurement of folic acid supplements during various gestational periods, including after 12 weeks of gestation, and measurements of epigenetic changes in offspring umbilical cord blood and methylation status of individual imprinted genes and repeat elements.	Increased consumption of folic acid supplements by pregnant women after 12 weeks of gestation was linked to epigenetic changes in fetal core blood, including increased methylation of the paternal copy of *IGF2* and decreased methylation of the paternally-imprinted *PEG3*, as well as repeat elements (LINE-1s).
Kim et al., 2014	A retrospective secondary analysis cohort population consisting of Korean mother and children dyads was examined. Pregnancies (215 included in the study) were delivered at Korea University Anam Hospital between July 1, 2009 and June 13, 2010. Maternal folate level was measured with an I^125^-based RIA.	Incidence of preeclamsia and small for gestational age births was reduced in folic acid supplemented group compared to control mothers.
Veeranki et al., 2014	A retrospective cohort study that included 167,333 women (15–44) and their biological infants born during 1995–2007 that were enrolled in the Tennessee Medicaid program (TennCare) was used for these studies. Infants were tracked for bronchiolitis in the first year of life.	Maternal supplementation of folic acid through prenatal vitamins beginning at the first trimester increased the likelihood of infant bronchiolitis
Dominguez-Salas et al., 2014	Gambian women and their children representing 24 villages and 166 women were included in this 2 years study that examined consumption of maternal methyl donors and DNA methylation pattern in the offspring genomes.	There was a strong association between maternal methyl donor supplementation and potentially harmful DNA methylation alterations in offspring genomes.
Schmidt et al., 2011	Study included children that ranged from 24 to 60 months of age (*N* = 288) and examined effects of maternal consumption of prenatal vitamins containing methyl components during the periconceptional period and offspring incidence of ASD.	Increased risk for autism spectrum disorders (ASD) was identified in children whose mothers did not consume prenatal vitamins supplemented with methyl components during the periconceptional period.
Schmidt et al., 2012	Measurement of folic acid intake during the periconceptional period and ASD risk in mothers and children, including those that had inefficient folate metabolism due to a single nucleotide polymorphism (SNP) in the methyhlenetetrahydrofolate reductase enzyme (MTHFR 677 C>T variant genotype).	Consumption of folic acid by women beginning at the periconceptional period was associated with reduced risk of ASD in mothers and children with inefficient folate metabolism.
Beard et al., 2011	Rochester Epidemiological Project, Rochester, MN study that spanned 1976–1997 and examined ASD incidence rates relative to maternal consumption of folic acid.	A strong correlation (0.87) was evident for maternal consumption of prescription prenatal vitamins (containing > 1 mg folic acid) and ASD incidence in offspring.

Other factors likely interact with maternal diet to influence the net effect of these diets. Examples include genetic background, sex, developmental windows of exposure, and tissue/cell-specific vulnerabilities, which are discussed below.

## Maternal methyl supplemented diets and outcomes in animal model studies

One of the first reports linking environmental exposure to epimutations suggested that for a specific genotype (*A^vy^/a* offspring), pregnant *a/a* female mice exposed to the endocrine disrupting compound (EDC) bisphenol A (BPA) gave birth to a greater percentage of yellow coat color compared to brown coat color (*A^vy^/a*) offspring (Dolinoy et al., 2007), although we were not able to reproduce these BPA-induced results (Rosenfeld et al., 2013). Nonetheless, this first report indicated that offspring of both BPA-exposed and control animals are genetically identical but differ epigenetically in the DNA methylation status in at least one allele, *A^vy^*, encoding for the agouti signaling protein (ASIP) (Dolinoy et al., 2007). Humans do not possess this allele, but ASIP is abundantly expressed in human adipose tissue and pancreas, where it has been linked with obesity and type 2 diabetes (Mynatt and Stephens, 2001; Voisey and Van Daal, 2002).

When the cryptic promoter derived from the retroviral intracisternal A particle (IAP) within this allele is hypomethylated, *A^vy^* is expressed ectopically and constitutively resulting in yellow coat color mice who are prone to developing these metabolic disorders. In contrast, if the IAP promoter remains methylated, it is non-functional, leading to pseudoagouti (brown) coat color animals that are concomitantly less prone to these adult diseases. Combined prenatal treatment with a BPA and methyl-supplemented diet in these females was reported to offset the BPA-induced shift in coat color distribution from metabolic deleterious yellow morphs to an increase in brown coat color, healthier offspring (Dolinoy et al., 2007). Earlier reports indicated that *a/a* females harboring *A^vy^/a* who were fed a methyl-enriched diet 2 weeks prior to conception through weaning gave birth to a greater percentage of brown coat color offspring (Wolff et al., 1998; Cooney et al., 2002; Waterland and Jirtle, 2003).

Methyl diet supplementation can interact with epigenotype (*A^vy^*/a status) over several generations (through F_5_) to increase percentage of pseudoagouti animals possessing a methylated IAP promoter site (Cropley et al., 2012). However, removal of MS in later generations led to reversal of this phenotypic distortion. While a MS diet did not increase 5-methylcytosine levels over six generations in C57Bl6J mice, this diet increased the stochastic epigenetic variation in several loci with effects becoming more pronounced in later generations on this diet (Li et al., 2011). Combined results suggest that MS diets lead to epigenetic variability over time and may account for altered disease risk, although no harmful multigenerational phenotypic effects were reported in C57Bl6J or *A^vy^/a* mice (Li et al., 2011; Cropley et al., 2012).

Mice deficient for twisted gastrulation (TWSG1), an extracellular modulator of bone morphogenetic protein signaling, exhibit midline facial and jaw defects. However, maternal MS diet prevented their jaw defects (Billington et al., 2013). Similarly, a maternal MS diet alleviated atherosclerosis in ApoE homozygous mutant mice and corresponding elevation of *Ccr2*, production of inflammatory cytokines, and restoration of the HDL to LDL ratio (Delaney et al., 2013). Maternal choline supplementation of mice has also been reported to abolish transcriptional defects in genes related to cell growth and metabolism and reduced weaning body weight that are typically observed in fetal toxic milk (tx-j) mice, a model of Wilson Disease (an autosomal recessive disorder leading to hepatic copper accumulation) (Medici et al., 2014). This maternal supplement may also improve spatial mapping and basal forebrain cholinergic neuron numbers in Ts65Dn mice (Ash et al., 2014).

Combined treatment of pregnant rodent dams with methyl supplements has been reported to mitigate harmful effects of a high fat diet, including metabolic, select behavioral, DNA methylation, and gene expression effects (Carlin et al., 2013). Maternal MS may also reverse alcohol-induced DNA methylation *Igf2* changes (Downing et al., 2011).

Maternal MS diet in rats resulted in decreased offspring concentration of leptin and hypermethylation of the leptin (*Ob*) promoter (Giudicelli et al., 2013). Both offspring sexes exhibited impaired post-natal growth, and males showed increased long-term body weight gain.

No animal model study to date, however, has considered the long-term impact of exposure to these supplements. Moreover, current diet recommendations are based on the premise that many diseases are triggered by hypomethylation of discrete CpG sites. However, only two animal models to date have linked epimutations in DNA methylation to maternal MS diet exposure: viable yellow mice (*A^vy^/a*) with metabolic disorders, discussed above, and *Axin^Fu^* mice exhibiting tail dysmorphogenesis, or “kinky” tail (Waterland et al., 2006; Rosenfeld, 2010). Both examples are the result of DNA methylation alteration in a specific gene-embedded retroviral particle, IAP. It is unlikely that consumption or maternal exposure to MS selectively targets a single locus within the genome. Instead, such nutritional supplements may induce widespread and promiscuous effects. The developing fetus is especially vulnerable to these changes, as this is when there is re-establishment of new epigenetic marks and programming of the primordial germ cells. It is thus not surprising that there are also mounting animal model studies reporting deleterious effects in offspring born to dams on a MS diet.

Combined exposure of mouse fetuses to arsenic and maternal MS, in the form of folate, led to more pronounced DNA methylation changes in the liver and corresponding impaired fetal development and body weight gain (Tsang et al., 2012). Maternal folic acid supplementation along with a high fat diet increased glucose intolerance and insulin resistance in male mice offspring (Huang et al., 2014). Another study in mice indicated that a maternal MS diet resulted in harmful and persistent changes in offspring gut microbiota populations, colonic mucosal DNA methylation, and gene expression changes, and increased incidence of colitis compared to control offspring (Schaible et al., 2011). A similar maternal dietary regimen increased the incidence of allergic airway disease in mice with corresponding hypermethylation of *Runx3*, a gene that suppresses allergic airway disease (Hollingsworth et al., 2008). This same maternal diet suppressed proinflammatory signals in mice (Delaney et al., 2012).

Treatment of rat dams fed a methyl donor, choline supplemented, or deficient diet resulted in selective fetal DNA methylation and histone protein marks (Davison et al., 2009). A maternal choline deficient diet resulted in hypermethylation of fetal *G9a* and *Suv39h1* relative to controls. Coincident with this hypermethylation, modified histones associated with transcriptional silencing, H3K9Me2 and H3K27Me3, increased with maternal choline supplementation. In contrast, the histone mark associated with active promoters, H3K4Me2, surged in expression with the choline-deficient maternal diet.

Gestational and postnatal exposure (in case of pup assessments) to high concentrations of folic acid resulted in newborn mouse pups exhibiting disrupted cerebral gene expression for transcripts and their protein products implicated in normal neural development (Barua et al., 2014). These maternal MS pups also exhibited behavioral alternations (increased ultrasonic vocalizations, heightened anxiety-like, and hyperactive behaviors).

## Human epidemiological studies linking maternal MS diets and offspring epimutations

Increased maternal intake during the periconceptional period of choline was inversely correlated with umbilical cord blood methylation of her sons (Boeke et al., 2012). Consumption of folic acid supplements by pregnant women after 12 weeks of gestation has been linked to epigenetic changes in fetal cord blood, including increased methylation of the paternally expressed *IGF2* gene and decreased methylation of the similarly imprinted *PEG3*, as well as LINE-1s (Haggarty et al., 2013). These results thus suggest that a maternal MS diet can influence expression of both repeat elements and imprinted genes in offspring. The long term phenotypic effects of such changes, however, remain unknown. In women, maternal folic acid supplementation has been linked to decreased incidence of preeclampsia and small for gestational age births (Kim et al., 2014). On the other hand, maternal supplementation of folic acid beginning at the first trimester increased the likelihood of infant bronchiolitis (Veeranki et al., 2014).

A recent study of Gambian women and their children demonstrated that consumption of a methyl enriched diet during pregnancy strongly correlated with persistent and systemic epigenetic changes in several metastable epialleles in offspring (Dominguez-Salas et al., 2014). This relatively large (24 villages, 166 women), 2 years study suggests a strong association between maternal methyl donor consumption and DNA methylation patterns in the genomes of offspring, which may ultimately result in adverse phenotypic outcomes.

## Linkage of maternal methyl supplemented diets and prevention/risk for ASD in her children

Although maternal supplementation with folic acid and other methyl donors contained within pre-natal vitamins has been promoted as a protection against Autism Spectrum Disorders (ASD) in children (Schmidt et al., 2011, 2012), other studies suggest an opposite correlation (Rogers, 2008; Leeming and Lucock, 2009; Beard et al., 2011). The study suggesting that maternal MS diets prevented ASD was based on a cohort population of 288 children that ranged from 24 to 60 months of age and found an associated greater risk for ASD in children of mothers who did not consume prenatal vitamins supplemented with methyl components during the periconceptional period (Schmidt et al., 2011). A follow-up study by this same group further suggested that consumption of folic acid by women beginning at the periconceptional period may reduce the risk for ASD in mothers and children with inefficient folate metabolism, i.e., a single nucleotide polymorphism (SNP) in methylenetetrahydrofolate reductase (MTHFR 677 C>T variant genotype). This enzyme catalyzes the conversion of 5,10-methylenetetrahydrofolate (5′,10,′-MTHF) to 5-methyltetrahydrofolate (5′-MTHF, Figure 1) and is thus the rate-limiting enzyme in the methyl cycle (Schmidt et al., 2012).

In contrast, a study that employed ASD incidence rates from the Rochester Epidemiological Project in Rochester, MN, which spanned decades from 1976–1997, suggested a strong correlation (0.87) for maternal consumption of prescription prenatal vitamins (containing > 1 mg of folic acid) and ASD incidence in offspring (Beard et al., 2011). Consequently, the investigators concluded that too much folic acid might disrupt brain development and thus increase the risk for ASD, a hypothesis promoted in previous studies (Rogers, 2008; Leeming and Lucock, 2009).

## Maternal methyl supplemented diets likely lead to systemic effects

Select animal model and human epidemiological studies highlight the primary concern with maternal and adult methyl supplemented diet regimens (Tables 1A and B). Methyl-enriched nutritional compounds mediate one-carbon metabolism (Figure 1) and thereby increase the availability of S-adenosylmethionine (SAM). SAM serves as the primary donor of methyl groups to a wide range of biomolecules, including nucleic acids and histone proteins. Therefore, it is not possible with this dietary approach to target specific biomolecules for prevention and remediation strategies. In some cases, these diets might induce hypermethylation and suppression of harmful sites, such as embedded retroelements and proto-oncogenes. However, tumor suppressor and other disease-suppressing genes might also become entrapped in this DNA methylation “net” and transcriptionally silenced. In other words, tinkering with methylation status on the global level, as likely occurs with consumption of methyl supplements, can affect both “beneficial” and “harmful” genes. The scale of disease promotion or prevention is likely tipped one way or another depending on the combined genes that are affected.

## Other factors influencing the epigenetic effects of maternal methyl supplemented diets

Genetic variation and other maternal factors may be important considerations in determining whether specific nutraceuticals are beneficial. It is clear that there are gene-nutrient interactions in one-carbon metabolism (Friso and Choi, 2005). As noted, maternal folate supplementation may help to reduce the incidence of ASD in mothers and children with a MTHFR 677 C>T variant genotype that leads to inefficient folate metabolism. In contrast, this same diet may increase the risk for ASD in those with normal folate metabolism (Rogers, 2008; Leeming and Lucock, 2009; Beard et al., 2011). Similarly, total intake of choline and maternal genotype in the MTHFR and phosphatidyl ethanolamine methyltransferase (PEMT) enzymes interact to alter the amount of choline and choline metabolites in the breast milk, and thereby the amount of total choline available to the developing infant (Fischer et al., 2010a). In mothers with genetic polymorphisms that alter the intake-metabolite concentrations, supplemental choline might be beneficial. Maternal estrogen concentrations and gut microbiota populations may also influence the requirement for choline supplementation (Fischer et al., 2010b; Spencer et al., 2011).

It is possible that not all methyl donors lead to similar epigenetic effects across target organs, tissues, and or cell types (as Reviewed in Ly et al., 2012). For example, diet duration and potential interactions with other dietary components may be confounding factors. While an in-depth comparison is not currently possible due to limited studies assessing individual methyl components, Table 1A provides details on the treatment strategies for the animal experiments detailed herein. As shown in this Table, considerable variations in concentrations of methyl donors alone and in combination with each other have been tested in the different rodent studies. Future experiments need to be designed to consider the safety of single nutrient vs. nutrient combinations and a range of doses for each supplement should be tested.

Concurrent exposure to other compounds may also influence the effects of a methyl supplemented diet. For instance, prenatal exposure to exogenous glucocorticoids alters placental transport, and subsequently the availability of methyl donors, with an increased transfer of folate; whereas, choline transport across this organ was suppressed (Wyrwoll et al., 2012). Correspondingly, reduced fetal plasma methionine levels were observed in the glucocorticoid exposed fetuses.

Sexually dimorphic differences might also occur in response to *in utero* or adult exposure to methyl supplemented diets, as has been reported for other diets (Mao et al., 2010). For example, both maternal folate supplementation and fetal sex influence the DNA methylation status of *Slc394a* in the intestinal tract (McKay et al., 2011). Similarly, maternal methyl-deficient diets selectively result in metabolic disturbances in male, but not female, rat offspring (Maloney et al., 2011). A diet deficient in key methyl donors (choline and methionine) has been linked with hepatic carcinogenesis in F344 male rats (Mikol et al., 1983; Wilson et al., 1984; Pogribny et al., 2006); whereas, these dietary deficiencies do not result in liver cancer in their female counterparts (Saito et al., 1991).

Lastly, methyl components may lead to tissue/cell-specific effects. Such differences would suggest that these diets might aide in disease prevention in select organs or tissues but also exacerbate disease risk in others. To our knowledge, however, there has not currently been a comprehensive examination of how a maternal MS diet affects varying tissues and somatic/germ cells, and thus impacted phenotypes. This information is critical in determining the global effects that might be anticipated in offspring born to mothers on these supplemented diets.

## Conclusions and future directions

The notion that “optimal” MS diets might be used for prevention and remediation strategies to treat various human diseases is gaining currency (Van Den Veyver, 2002). Mounting studies have reported on effects of maternal MS diets in animal models and human epidemiological studies. However, there is currently no consensus on whether these dietary regimens are actually protective against a range of diseases. While early reports suggested that these diets might be useful in prevention strategies in epigenetic and transgenic animal models (Dolinoy et al., 2007; Billington et al., 2013; Delaney et al., 2013; Giudicelli et al., 2013), subsequent studies in animal models have suggested that maternal MS diets result in widespread epimutations that may govern diverse diseases (Hollingsworth et al., 2008; Schaible et al., 2011; Tsang et al., 2012). Additionally, maternal MS diets may alter the DNA methylation of a myriad of genes, including repetitive elements and imprinted genes in humans (Haggarty et al., 2013; Dominguez-Salas et al., 2014). The extent of such epimutations, and their biological consequences, remain to be determined.

While the idea of an “optimal” maternal MS diet preventative/treatment strategy is appealing, this diet approach cannot be used to target selectively a single gene. Instead, indiscriminate and potentially irreversible methylation changes in a broader range of biomolecules and cells/tissues may result from such dietary treatment. Potential disease outcomes are likely tipped one way or another depending on the fetal sex, maternal/fetal genetic background, other maternal factors (such as endogenous and exogenous circulating hormones and the intestinal microbiome), as well as tissue/cell specificity and concentration and duration of exposure to these nutritional supplements. All of these factors should be considered when employing maternal MS diets to combat fetal disease.

Comprehensive long term animal and human epidemiological studies should be undertaken to assess the global effects of these nutraceuticals and to resolve the current conflicting data in regards to maternal MS diet. Until then, women requesting prenatal/perinatal vitamin support should be advised against consuming methyl supplements, (e.g., folic acid) in doses that exceed the current recommendations for preventing fetal neural tube defects.

### Conflict of interest statement

The authors declare that the research was conducted in the absence of any commercial or financial relationships that could be construed as a potential conflict of interest.
